# COVID-19 Vaccination Willingness in Four Asian Countries: A Comparative Study including Thailand, Indonesia, the Philippines, and Vietnam

**DOI:** 10.3390/ijerph191912284

**Published:** 2022-09-27

**Authors:** Kiyoko Saito, Makiko Komasawa, Myo Nyein Aung, Ei Thinzar Khin

**Affiliations:** 1 JICA Ogata Sadako Research Institute for Peace and Development, Japan International Cooperation Agency (JICA), Tokyo 169-8433, Japan; 2 Department of Global Health Research, Graduate School of Medicine, Juntendo University, Tokyo 113-8421, Japan

**Keywords:** COVID-19 vaccines, willingness, Thailand, Indonesia, Philippines, Vietnam

## Abstract

Globally, 67% of the population has received at least one COVID-19 vaccine dose, but coverage varies across countries. This study aimed to compare people’s willingness to receive COVID-19 vaccination across Thailand, Indonesia, Philippines, and Vietnam, where vaccination intention tends to be high, to determine factors associated with willingness, and to obtain suggestions for developing strategies. We conducted a secondary data analysis of the Japan International Cooperation Agency survey “Building Resilience: COVID-19 Impact and Responses in Urban Areas—Case of Southeast Asia,” including1842 unvaccinated participants from Thailand (*n* = 461), Indonesia (*n* = 246), the Philippines (*n* = 609), and Vietnam (*n* = 526). Vaccination willingness was high in all countries (69.6%), but the social and psychological factors motivating people to undergo vaccination differed among these countries. The highest vaccination willingness was in the Philippines, followed by Vietnam and Indonesia, whereas the lowest vaccination willingness was in Thailand. Vaccination willingness was affected by not only sociodemographic attributes, but also risk perception and beliefs, which, in turn, were shaped by social factors such as infection trends and vaccine policies. To achieve effective vaccination promotion programs, a system allowing the flexible modification of promotion methods in response to social conditions must be established.

## 1. Introduction

Considering the characteristics of the COVID-19 virus, including high infectivity and severity, and the impact on daily life resulting from social distancing and business interruption, vaccination is the key. Globally, 67% of the population has received at least one vaccine dose, but coverage varies between countries [1]. East and Southeast Asia showed relatively high acceptance [2]. Another study also found high acceptance results in South America but low results in the Middle East and North Africa [3,4], as well as in Europe, Central Asia, and West and Central Africa [4]. In a survey comparing upper-middle-income and lower-middle-income country groups, a trend toward stronger vaccination intentions was noted in lower-middle-income countries [5]. Additionally, Asians were most willing to be vaccinated, followed by non-Hispanic whites, Hispanics, and non-Hispanic blacks [6]. The WHO states that immunization efforts must be expanded to include diverse populations, both national and international, who were typically not vaccinated in the past, and that strategic efforts to expand immunization are needed to first identify factors that promote such expansion and then strategically introduce interventions, or combinations of interventions, according to these factors [7].

Many factors have been associated with vaccination intention [2,6,8,9,10,11]. A review study of peer-reviewed articles divided factors influencing intention to undergo COVID-19 vaccination into seven categories (demographics, social factors, beliefs and attitudes about vaccination, vaccine perceptions, health perceptions, barrier perceptions, and vaccine recommendations) [2,12,13,14], which may differ across countries. Other factors include a sense of purpose [15], self-efficacy, perceived divine will, perceived action efficacy of the COVID-19 vaccines, and action efficacy of the vaccine to control virus spread [9,16].

To establish an effective strategy, we should consider the factors that influence vaccination behavior and the background that causes these factors as the foundation for developing a tailor-made strategy, which varies from country to country.

Therefore, this study conducted a comparative analysis of vaccination willingness and its factors in four Asian countries where vaccination intention tends to be high and to obtain suggestions on how to develop vaccination-promoting strategies.

## 2. Materials and Methods

We conducted a secondary data analysis of the Japan International Cooperation Agency (JICA) survey “Building Resilience: COVID-19 Impact and Responses in Urban Areas” [17], which was the behavioral change survey in the COVID-19 pandemic. The survey was uploaded and administered via an online survey platform and was open to registered people from four countries, namely, Thailand, Indonesia, the Philippines, and Vietnam. From the end of April to May 2021, a link to the electronic survey was distributed via various methods, including invitations via email and social media platforms. A statement of informed consent was provided on the first page of the questionnaire. All the participants’ responses remained anonymous according to JICA’s privacy policy.

Survey questionnaire information was collected on participants’ demographic characteristics, socioeconomic status, and occupation. Questions used in the secondary analysis were segregated into the following three parts:

1. *Risk perception of the COVID-19 pandemic.* The items were subdivided into three components: (a) Confidence in knowledge, (b) Concerns about health risk, and (c) Concerns about economic well-being. Questions were answered using a five-scale method, which was categorized into three to improve the efficiency of the analysis: Strongly and Somewhat likely, Neither Agree nor Disagree, and Strongly and Somewhat agree. Table 1 lists the items included in each component.
2. *Willingness to receive the vaccine*. This part was assessed by addressing one question item: “In your current situation, how likely are you to make an appointment, then go and get vaccinated for COVID-19?” Willingness to receive the COVID-19 vaccine was responded to by a five-point scale, which was combined into three categories: Highly and Somewhat likely, Maybe, Somewhat, and Highly unlikely.
3. *Reasons (factors) to take the vaccine.* The factors were subdivided into positive and negative factors. Respondents were asked to select the top three positive and negative factors that would influence their decision to undergo vaccination. Positive response options comprised 16 items, whereas negative response options comprised 17 items. Table 2 lists these items.

### Statistical Analysis

The data used in the analysis were categorical variables, which were examined using Pearson’s chi-square test and are expressed as absolute values (percentages).

The independent predictors of COVID-19 vaccination willingness among people from the four selected Asian countries were evaluated using multivariate logistic regression. A value of *p* < 0.05 was considered significant. All statistical data were analyzed using STATA for Windows, Version 17, StataCorp., College Station, TX, USA.

## 3. Results

### 3.1. Participants

The purpose of this study was to perform a comparative analysis of willingness to vaccinate and to determine contributing factors; therefore, the targeted respondents were those who had not yet been vaccinated and would be vaccinated in the future.

Of 2684 respondents from the four Asian countries, 1842 (Thailand, 461; Indonesia, 246; the Philippines, 609; and Vietnam, 526) were not vaccinated. The sex ratio of this unvaccinated population was closely comparable across these four countries (% of males: Thailand, 49.2%; Indonesia, 52.8%; the Philippines, 51.4%; Vietnam, 51.7%); the mean ages of this population in Thailand, Indonesia, the Philippines, and Vietnam were 35.6 (SD 10.9), 34.2 (SD 10.9), 32.1 (SD 9.6), and 31.1 (SD 8.7) years, respectively. Table 3 presents the sociodemographic characteristics of the unvaccinated participants. 

### 3.2. Risk Perception of the COVID-19 Pandemic 

Table 4 presents the frequency of “Strongly & somewhat disagree”, “Neither agree nor disagree” and “Strongly & somewhat agree”. 

#### 3.2.1. Confidence in Knowledge

Participants from Vietnam were the most confident in their knowledge (88.4%), whereas those from Indonesia tended to be significantly less confident than the other countries (70.7%, *p* < 0.001). 

#### 3.2.2. Concerns about COVID-19 Health Risk

More than half of all respondents in all countries perceived the risk of COVID-19 death to be high, with Vietnam taking it more seriously (84.6%, *p* < 0.001) and considering it different from the regular flu (69.2%). All people from all selected countries were aware of the high risks, especially for older relatives and parents; among these countries, Indonesia was the least aware (76.0%, *p* < 0.001).

Many participants believed that the world is in serious danger because of COVID-19. In particular, 93.7% of Vietnamese perceived the risk severely. However, despite the COVID-19 risk and the serious situation worldwide, they tended to be less aware of themselves. Vietnam, where the largest number of participants considered the world situation serious, had the highest number of participants who considered themselves safe (52.1%). However, contrary to the trend in other countries, more than half of the participants from Thailand believed that they were unsafe (72.0%, *p* < 0.001).

#### 3.2.3. Concerns about Economic Well-Being

More than half of the participants were concerned about the financial crisis and did not feel financially secure. Thailand was the most concerned about these two aspects (81.8% and 83.5%, respectively, *p* < 0.001 for both), whereas Indonesia appeared relatively optimistic compared with other countries (56.1% and 62.2%, respectively, *p* < 0.001 for both).

### 3.3. Willingness to Receive Vaccinations

More than half of the unvaccinated participants from all countries expressed willingness to receive the COVID-19 vaccine, with the highest number of willing participants in the Philippines (75.7%, *p* < 0.001), as shown in Table 5.

### 3.4. Reasons (Factors) to Receive Vaccination

#### 3.4.1. Positive Factors

The most selected positive factor for receiving vaccination was “Vaccines will keep my loved ones safe from COVID-19” in all countries, except for Vietnam (Thailand, 43.8%; Indonesia, 40.6%; and the Philippines, 55.5%). Other major positive factors included “I am afraid of getting COVID-19,” “Vaccines are safe and effective,” “Experts say it is safe and effective,” and “It’s free”.

Apart from “Vaccines will keep my loved ones safe from COVID-19” as the most selected factor, “It’s free” (38.4%) and “Vaccines are safe and effective” (34.3%) were the second and third most selected factors in Thailand; “Experts say it is safe and effective” (32.1%), and “Vaccines are safe and effective” (30.9%) in Indonesia; and “I am afraid of getting COVID-19” (42.0%) and “Experts say it is safe and effective” (38.3%) in the Philippines. Those in Vietnam mostly selected “Vaccines are safe and effective” (52.3%), followed by “I am afraid of getting COVID-19” (52.1%) and “Vaccines will keep my loved ones safe from COVID-19” (44.1%). Table 6 presents the frequency of selection of positive factors in each country.

#### 3.4.2. Negative Factors

Overall, the most selected negative factor was “Possible side effects” in all countries (Thailand, 75.9%; Indonesia, 53.3%; the Philippines, 73.1%; and Vietnam, 55.5%). Other major negative factors were “Vaccines are not safe or effective in general,” “Not enough evidence that it prevents COVID-19,” “I do not know when and where to get it,” and “Vaccines are not safe or effective in general.”

Regarding the results in each country, in Thailand, the second and third most selected factors were “Vaccines are not safe or effective in general” (62.3%) and “Not enough evidence that it prevents COVID-19” (34.9%). Indonesia and Vietnam showed the same trend, with “I don’t know when and where to get the vaccine” (Indonesia, 27.2% and Vietnam, 29.3%) as the second most selected factor and “Not enough evidence that it prevents COVID-19” (Indonesia, 24.0% and Vietnam, 24.3%) as the third. In the Philippines, “Not enough evidence that it prevents COVID-19” (36.9%) was the second most selected factor, and “Approvals and clinical trials were too fast” (27.9%) was the third. Table 7 presents these results.

### 3.5. Independent Predictors of Vaccination Willingness

Independent predictors of willingness to receive vaccination were assessed by multivariate analysis. After adjusting for the important covariates, the predictors of vaccination willingness included the index of sociodemographic attributes, risk perception of COVID-19, and beliefs and attitudes toward vaccination. Below are the results of each index.

#### 3.5.1. Sociodemographic Attribute Index

Indonesia was the only country that regarded (older) age as an increased index for the predictors of vaccination willingness (adjusted odds ratio (AOR), 2.35; 95% confidence interval (CI), 1.20–4.59; *p* < 0.05).

Other increased indexes were (decreased) were:

- Income in Indonesia (AOR, 1.97; 95% CI, 1.08–3.60, *p* < 0.05);
- (higher) Education in Thailand (AOR, 2.25; 95% CI, 1.31–3.87; *p* < 0.05) and Indonesia (AOR, 2.46; 95% CI, 1.30–4.67; *p* < 0.05);
- Work style, particularly retired or homemakers in Thailand (AOR, 5.19; 95% CI, 1.30–20.65; *p* < 0.05);
- Full-time or business owners in Vietnam (AOR, 4.43; 95% CI, 1.30–15.15; *p* < 0.05).

Conversely, (more) multigenerational living (AOR, 0.39; 95% CI, 0.20–0.78; *p* < 0.05) and (older) age (AOR, 0.50; 95% CI, 0.31–0.78; *p* < 0.05) in Vietnam were the only decreased indexes. The Philippines did not have any factor affecting vaccination willingness. Table 8 shows these results.

#### 3.5.2. Perception of the COVID-19 Pandemic

In Indonesia, the increased index was health risk perception, including “The world is in serious danger due to the COVID-19” (AOR, 8.28; 95% CI, 2.23–30.73; *p* < 0.05) and “Most concern about older relatives/parents” (AOR, 4.47; 95% CI, 1.28–15.59; *p* < 0.05).

In the Philippines, the health risk perception, including “People of all ages are dying from the COVID-19” (AOR, 1.77; 95% CI, 1.00–3.13; *p* < 0.05) and “The world is in serious danger due to the COVID-19” (AOR, 3.22; 95% CI, 1.38–7.53, *p* < 0.05), was the increased index. Table 9 presents these results. There were no factors affecting vaccination willingness in Thailand and Vietnam.

#### 3.5.3. Reasons for Vaccinations

##### Positive Factors

In all countries, except Vietnam, “Vaccine will keep my loved ones safe from COVID-19” was an increased index (Thailand (AOR, 2.49; 95% CI, 1.64–3.80; *p* < 0.001), Indonesia (AOR, 5.09; 95% CI, 2.47–10.49; *p* < 0.001), and the Philippines (AOR, 1.65; 95% CI, 1.12–2.41; *p* < 0.05)), whereas “It’s free” was a decreased index (Thailand (AOR, 0.57; 95% CI, 0.37– 0.86, *p* < 0.01), Indonesia (AOR, 0.46; 95% CI, 0.24–0.88; *p* < 0.05), and the Philippines (AOR, 0.64; 95% CI, 0.43–0.97; *p* < 0.05)). Moreover, “Enough time has passed since people started taking it” was a decreased index in Thailand (AOR, 0.34; 95% CI, 0.18–0.67; *p* < 0.01) and Vietnam (AOR, 0.32; 95% CI, 0.14–0.75; *p* < 0.01) but an increased index in Indonesia (AOR, 4.27; 95% CI, 1.16–15.67; *p* < 0.05). The Philippines had a decreased index for “My employer requires me to take it” (AOR 0.45, 95% CI, 0.27–0.75, *p* < 0.01), which, in other countries, did not affect vaccination willingness. “Experts say it is safe and effective” was a decreased index only in Thailand (AOR, 0.61, 95% CI, 0.38–0.98, *p* < 0.05). Table 10 enumerates these results.

##### Negative Factors

“I do not have the ability to get an appointment” was an increased index in Thailand (AOR, 3.85; 95% CI, 1.61–9.20; *p* < 0.01) and the Philippines (AOR, 3.36; 95% CI, 1.49–7.59, *p* < 0.01). Only Vietnam included “It contained human stem cells in its development” as an increased index (AOR, 3.92; 95% CI, 1.60–9.60; *p* < 0.01). “Approvals and clinical trials were too fast” (AOR, 2.11; 95% CI, 1.11–4.03, *p* < 0.05) and “I am concerned it will impact my fertility or pregnancy” (AOR, 2.22; 95% CI, 1.03–4.76; *p* < 0.05) were increased indexes in Thailand. Thailand and the Philippines considered “Not enough evidence that it prevents COVID-19” (AOR, 0.54; 95% CI, 0.36–0.83; *p* < 0.01 and AOR, 0.58; 95% CI, 0.39–0.85; *p* < 0.01, respectively) and “Vaccines are not safe or effective in general” (AOR, 0.43; 95% CI, 0.28–0.66; *p* < 0.001 and AOR, 0.57; 95% CI, 0.36–0.90; *p* < 0.05, respectively) as decreased indexes. Vietnam was also the only country that included “Possible side effects” as a decreased index (AOR, 0.53; 95% CI, 0.34–0.80, *p* < 0.01), whereas Indonesia was the only country that included “I do not trust the sites where vaccines are being administered” (AOR 0.25, 95% CI, 0.10–0.62, *p* < 0.01) as a decreased index. Table 11 lists these results.

## 4. Discussion

Our study revealed complex social and psychological factors that influence people’s willingness to receive COVID-19 vaccination in four selected countries. COVID-19 risk perception and vaccination willingness were high in all countries (total = 69.6%), similar to previous results that were around 60–70% [6,18,19]. However, even with the same high-risk perception and vaccination willingness, the social and psychological factors that motivate vaccination differed between countries. Vaccination willingness was most frequent in the Philippines, followed by Vietnam and Indonesia, and Thailand was the least frequent.

Our analysis of the characteristic trends in social and psychological factors in the Philippines revealed the following: (1) sociodemographic factors did not affect vaccination willingness; (2) a more serious risk perception, including death risk for all generations, made people more willing to be vaccinated; and (3) the abovementioned altruistic factor and the fear of COVID-19 infection were driving the motivation of highly willing people to undergo vaccination. Meanwhile, factors not directly related to vaccine effectiveness, such as cost and employer demands, did not affect highly willing people but affected the unwilling ones. The inability to make an appointment was the only factor demotivating people with high willingness. Alternatively, the lack of evidence supporting the vaccine’s effectiveness was a demotivating factor for unwilling people. People’s vaccination intent being affected by the perceptions of high susceptibility and severity is consistent with the results of previous studies.

Second, people from Indonesia perceived that education and decreased income affected their willingness to be vaccinated. According to other studies, people with a bachelor’s degree or higher were more likely to be vaccinated because they understood the vaccine’s efficacy and safety, and those in the middle class were vaccinated to keep working and earn money [20]. Age, education, and income had a strong effect on vaccination willingness. Additionally, participants from Indonesia who perceived the COVID-19 risk as a serious global risk factor were more strongly willing to be vaccinated than those from other countries. Indonesia was the only country where concerns for older relatives/parents affected vaccination willingness. Furthermore, keeping loved ones safe was the most motivating factor for vaccination among the three countries except Vietnam. Time was also a strong motivating factor for those who expressed willingness for vaccination. Distrust of the sites where vaccines were beginning to be administered was the only demotivating factor; however, free-of-charge vaccination was the only motivating factor for unwilling people.

In Vietnam, the following findings were obtained. First, people with higher incomes and those who worked as full-time employees or business owners were more willing to be vaccinated, whereas multigenerational families were less willing. Second, no sociodemographic factor affected vaccination willingness. Third, concern regarding the use of human stem cells was the only demotivating factor among highly willing people, whereas side effects were only the demotivating factor for the unwilling ones. Only participants in Vietnam considered that the altruistic factor did not affect vaccination willingness, similar to other studies in which such willingness was not influenced by family members, friends, or colleagues [21]. Compared to the other countries, fewer factors influenced vaccination willingness in Vietnam.

Finally, in Thailand, as reported in other Thai surveys [22], (1) education and employment status affected vaccination willingness, (2) perception of the COVID-19 pandemic did not affect vaccination willingness, and (3) the altruistic factor of keeping loved ones safe from COVID-19 motivated vaccination. The most demotivating factor for people positive about vaccination was the lack of ability to make an appointment. Although they had a slight concern regarding the vaccine’s effectiveness and safety and believe that the COVID-19 vaccination is supported by evidence, they have concerns about the premature approval process of the COVID-19 vaccine and pregnancy, leading to feelings of not being completely comfortable with this vaccine. For unwilling people, concerns about vaccine efficacy and safety were their demotivating factors.

**Figure 1:**
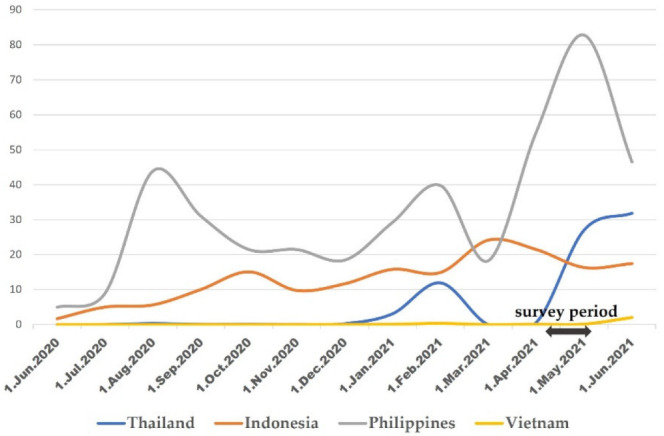
Number of new infections.

Evaluation of the differences among the countries suggests that the infection status affected the social and psychological factors motivating or demotivating people from receiving the vaccination. The number of infections and the rate of increase were high in the Philippines, whereas the number of infections was stably high with a low rate of increase in Indonesia. Alternatively, the number of infections was low in both Thailand and Vietnam, although the rate of increase was high in Thailand but low in Vietnam (Figure 1). In the Philippines, where both the number of infections and the rate of increase were very high compared to other countries, willingness to be vaccinated tended to be high. In the Philippines, people perceived that the risk of COVID-19 was more serious compared to the other countries, and doubts about the efficacy and safety of the vaccine did not demotivate vaccination. Some unwilling people who strongly doubt the efficacy and safety of the vaccination remained but were likely motivated by external factors such as demands from employers and cost-free vaccination.

Similarly, the trends in Indonesia, where the number of infections was high and the rate of increase was not, differed from the trends in the Philippines. People were becoming more confident that their communities had the infection under control. Concerns about the elderly individuals’ risk of COVID-19 might have reinforced people’s desire for control and motivation for vaccination. The increase in “enough time has passed since the start of vaccination” as a predictor of high willingness to vaccinate might be due to the fact that Indonesia started the COVID-19 vaccination the earliest among the four countries (January 2021), whereas the other three countries started the vaccination in February 2021 or later.

In Vietnam, which had the lowest number of infections and the lowest rate of increase, people were not aware that the risk of COVID-19 was a threat and were not in a situation where they were forced to choose their vaccination behavior, which resulted in few factors affecting their willingness of vaccination.

In Thailand, the number of infections was as low as that observed in Vietnam, but the rate of increase was higher. It is considered that, unlike in Vietnam, people in Thailand were beginning to feel closer to the threat of COVID-19. Similar to that observed in Vietnam, there were strong safety and efficacy concerns in Thailand, but the increase in infection numbers forced the public to make decisions about their vaccination behavior, resulting in a clear differentiation in the attitudes and beliefs of people who were willing and those who were not willing to vaccinate. The differentiation was reflected in the number of more predictors of willingness to be vaccinated than in Vietnam. Furthermore, the factors that contributed to the high rate of doubts about the safety and efficacy of vaccines among people who were unwilling to vaccinate were identical to those found in the Philippines, where the rate of increase was also high. Thus, even though the threat of infection was close and the sense of fear was amplified, unwilling people perceived more risk for efficacy and safety than for getting infected with COVID-19. Thus, for those with negative intentions towards vaccination, doubts about safety and efficacy might be viewed as a factor that is less susceptible to the infection status. 

## 5. Conclusions

Vaccination willingness was affected not only by sociodemographic attributes, but also by risk perception and beliefs, which, in turn, were shaped by social factors such as daily changing infection trends and vaccine policies. This result might reflect that most risk perceptions and beliefs were susceptible to external and dynamic circumstances. On the other hand, doubts about safety and efficacy were less sensitive to the infection, which indicates that the factors were robust and that resolving concerns and promoting behavioral changes might be quite difficult. Therefore, it is necessary to establish a system that not only evaluates risks from public health and socioeconomic perspectives and allows for flexible changes in promotion methods based on assessment results, but also ensures that information on vaccine safety and efficacy, along with accurate scientific evidence, is disclosed in a stable and consistent manner.

Therefore, establishing a system combining flexibility in response to public health and social–economic status and continuous consistency with accurate information on vaccine safety and efficacy will make vaccination-promoting programs effective.

## 6. Limitations

First, this is a secondary analysis aimed at analyzing behavior change during the COVID-19 pandemic; hence, a limitation exists in analyzing precisely vaccination willingness. Second, this study involved an online survey by registrants; thus, a bias in the participants is possible, thereby limiting the versatility of the results. Bias factors include education and employment status. Tertiary and above registrants were overwhelmingly higher than the secondary registrants, which did not differ among the countries (Thailand, 78.7%; Indonesia, 72.9%; the Philippines, 86.5%; Vietnam, 88.6%). Additionally, the rate of part-time or business registrants was higher than the unemployed, retired/homemaker, and part-time registrants (Thailand, 69.0%; Indonesia, 61.5%; the Philippines, 62.0%; Vietnam, 72.0%), which may be due to the participation of registrants with computer literacy in the online survey. Third, this analysis was aimed at vaccination willingness; hence, only those data from unvaccinated people were selected, resulting in a small number of data being analyzed. Therefore, future studies with a larger sample size should be conducted according to our results, focusing on the relationship between infection status and government COVID-19 measures and vaccination behavior.

## Figures and Tables

**Table 1:** Questions for risk perception of the COVID-19 pandemic.

Question Items	
**Confidence in Knowledge**	
	I am well read and know a lot about the coronavirus
**Concerns about health risk**	
	The world is in serious danger due to the coronavirus
	The coronavirus is similar to the regular flu and has similar death rates
	People of all ages are dying from the coronavirus
	I am safe from the virus where I live, but not the rest of world
	Most concerned about older relatives/parents
**Concerns about economic well-being.**	
	Current pandemic economic is worse than that of Global Financial Crisis of 2008
	I am not financially secure because of the pandemic

**Table 2:** Questions for reasons (factors) to receive vaccination.

Positive	Negative
**Enabling environment**	**Enabling environment**
It’s free	I do not know when and where to get it
Vaccine site is convenient/nearby	I do not have the ability to get an appointment
It’s easy to make an appointment	I cannot access an appointment time outside of my working hours
My employer provides paid time off to take it	I do not trust the sites where vaccines are being administered
**Vaccine effect and safety**	**Vaccine effect and safety**
Vaccines are safe and effective	Possible side-effects
Enough time has passed since people started taking it	Not enough evidence that it prevents COVID 19
Experts say it is safe and effective	Approvals and clinical trials were too fast
Recommended by a physician/doctor	Not enough time since people started taking it
My loved ones recommended that I take it	Not enough people have taken the vaccine
People I know took it	Vaccines are not safe or effective in general
Leaders I trust took it	Storage of the vaccine is concerning
**Risk Aversion**	It contained human stem cells in its development
I’m afraid of getting COVID 19	Clinical trials did not include adequate diversity or enough
Vaccines will keep my loved ones safe from COVID 19	I am concerned it will impact my fertility or pregnancy
**Lifestyle**	**Risk Aversion**
To return to my previous lifestyle	I’m not afraid of getting COVID 19
To return to work/school	I already had COVID 19
**Social Norm**	Religious concerns
My employer requires me to take it	

**Table 3:** Sociodemographic characteristics of the participants (have not yet been vaccinated).

		Thailand (*n* = 461) *n* (%)	Indonesia *(n* = 246) *n* (%)	Philippines (*n* = 609) *n* (%)	Vietnam (*n* = 526) *n* (%)	*x* ^2^	*p*
**Age**							
	<35	193 (41.9)	152 (61.8)	413 (67.8)	372 (70.7)	104.05	<0.001
	≥35	268 (58.1)	94 (38.2)	196 (32.2)	154 (29.3)		
**Sex**							
	Male	227 (49.2)	130 (52.8)	313 (51.4)	272 (51.7)	1.04	0.79
	Female	234 (50.8)	116 (47.2)	296 (48.6)	254 (48.3)		
**Education**							
	Up to secondary	73 (15.8)	77 (31.3)	50 (8.2)	42 (8.0)	118.76	<0.001
	Tertiary and above	386 (83.7)	169 (68.7)	538 (88.3)	478 (90.9)		
	Others	2 (0.5)	0 (0.0)	21 (3.5)	6 (1.1)		
**Family type**							
	Living alone	68 (14.7)	21 (8.5)	50 (8.2)	60 (11.4)	32.72	<0.001
	Living with friends/partner/spouse with or without children	224 (48.6)	158 (64.3)	314 (51.6)	303 (57.6)		
	Multi-generation	169 (36.7)	67 (27.2)	245 (40.2)	163 (31.0)		
**Employment**							
	Un-employed	25 (5.4)	17 (6.9)	53 (8.7)	14 (2.7)	81.43	<0.001
	Retired or homemaker	25 (5.4)	10 (4.1)	24 (3.9)	5 (0.9)		
	Part-time	79 (17.2)	92 (37.4)	117 (19.2)	113 (21.5)		
	Full-time or business	332 (72.0)	127 (51.6)	415 (68.2)	394 (74.9)		
**Monthly household income**							
	Below average	127 (27.6)	122 (49.6)	435 (71.4)	98 (18.6)	480.96	<0.001
	Average	158 (34.3)	50 (20.3)	83 (13.6)	82 (15.6)		
	Above average	176 (38.2)	74 (30.1)	91 (14.9)	346 (65.8)		
**Income decreased due to COVID-19**							
	Yes	307 (66.6)	154 (62.6)	338 (55.5)	342 (65.0)	17.11	0.001
	No	154 (33.4)	92 (37.4)	271 (44.5)	184 (35.0)		

**Table 4:** Perception about the COVID-19 of the participants not vaccinated yet (frequency).

		Thailand (*n* = 461) *n* (%)	Indonesia (*n* = 246) *n* (%)	Philippines (*n* = 609) *n* (%)	Vietnam (*n* = 526) *n* (%)	*x* ^2^	*p*
**Knowledge about COVID-19**							
**I am well read and know a lot about the coronavirus**							
	Strongly & somewhat disagree	29 (6.3)	18 (7.3)	85 (14.0)	24 (4.6)	95.68	<0.001
	Neither agree nor disagree	29 (6.3)	54 (22.0)	47 (7.7)	37 (7.0)		
	Strongly & somewhat agree	403 (87.4)	174 (70.7)	477 (78.3)	465 (88.4)		
**Concerns about COVID-19**							
**The coronavirus is similar to the regular flu and has similar death rates**							
	Strongly & somewhat disagree	270 (58.6)	108 (43.9)	290 (47.6)	364 (69.2)	76.69	<0.001
	Neither agree nor disagree	48 (10.4)	41 (16.7)	76 (12.5)	56 (10.7)		
	Strongly & somewhat agree	143 (31.0)	97 (39.4)	243 (39.9)	106 (20.1)		
**People of all ages are dying from the coronavirus**							
	Strongly & somewhat disagree	121 (26.2)	39 (15.9)	91 (14.9)	37 (7.0)	129.92	<0.001
	Neither agree nor disagree	72 (15.6)	53 (21.5)	44 (7.3)	44 (8.4)		
	Strongly & somewhat agree	268 (58.1)	154 (62.6)	474 (77.8)	445 (84.6)		
**The world is in serious danger due to the coronavirus**							
	Strongly & somewhat disagree	27 (5.8)	20 (8.1)	32 (5.2)	13 (2.5)	49.78	<0.001
	Neither agree nor disagree	28 (6.1)	36 (14.7)	34 (5.6)	20 (3.8)		
	Strongly & somewhat agree	406 (88.1)	190 (77.2)	543 (89.2)	493 (93.7)		
**I am safe from the virus where I live, but not the rest of world**							
	Strongly & somewhat agree	91 (19.7)	108 (43.9)	220 (36.1)	274 (52.1)	231.91	<0.001
	Neither agree nor disagree	38 (8.3)	66 (26.8)	134 (22.0)	98 (18.6)		
	Strongly & somewhat disagree	332 (72.0)	72 (29.3)	255 (41.9)	154 (29.3)		
**Most concerned about older relatives/parents**							
	Strongly & somewhat disagree	24 (5.2)	17 (6.9)	52 (8.6)	28 (5.3)	71.15	<0.001
	Neither agree nor disagree	27 (5.9)	42 (17.1)	13 (2.1)	52 (9.9)		
	Strongly & somewhat agree	410 (88.9)	187 (76.0)	544 (89.3)	446 (84.8)		
**Concerns about economic well-being**							
**Current pandemic economic is worse than that of Global Financial Crisis of 2008**							
	Strongly & somewhat disagree	26 (5.6)	49 (19.9)	43 (7.1)	49 (9.3)	83.09	<0.001
	Neither agree nor disagree	58 (12.6)	59 (24.0)	107 (17.6)	132 (25.1)		
	Strongly & somewhat agree	377 (81.8)	138 (56.1)	459 (75.31)	345 (65.6)		
**I am not financially secure because of the pandemic**							
	Strongly & somewhat disagree	30 (6.5)	35 (14.2)	95 (15.6)	84 (16.0)	113.82	<0.001
	Neither agree nor disagree	46 (10.0	58 (23.6)	96 (15.8)	159 (30.2)		
	Strongly & somewhat agree	385 (83.5)	153 (62.2)	418 (68.6)	283 (53.8)		

**Table 5:** COVID-19 Vaccination Characteristics.

		**Thailand** **(*n* = 1010)** ***n* (%)**	**Indonesia** **(*n* = 1018)** ***n* (%)**	**Philippines** **(*n* = 1012)** ***n* (%)**	**Vietnam** **(*n* = 1032)** ***n* (%)**	**Total** **(*n* = 4072)** ***n* (%)**	***x* ^2^**	***p***
**Eligible for the COVID19 vaccine**								
	Yes	605 (59.9)	668 (65.6)	690 (68.2)	721 (69.9)	2684 (65.9)	69.48	<0.001
	No	158 (15.6)	183 (18.0)	92 (9.1)	104 (10.1)	537 (13.2)		
	Don’t know	247 (24.5)	167 (16.4)	230 (22.7)	207 (20.0)	851 (20.9)		
		**Thailand** **(*n* = 605)** ***n* (%)**	**Indonesia** **(*n* = 668)** ***n* (%)**	**Philippines** **(*n* = 690)** ***n* (%)**	**Vietnam** **(*n* = 721)** ***n* (%)**	**Total** **(*n* = 2684)** ***n* (%)**	***x^2^***	***p***
**Already vaccinated**								
	Yes	144 (23.8)	422 (63.2)	81 (11.7)	195 (27.1)	842 (31.4)	459.69	<0.001
	No	461 (76.2)	246 (36.8)	609 (88.3)	526 (72.9)	1842 (68.6)		
		**Thailand** **(*n* = 461)** ***n* (%)**	**Indonesia** **(*n* = 246)** ***n* (%)**	**Philippines** **(*n* = 609)** ***n* (%)**	**Vietnam** **(*n* = 526)** ***n* (%)**	**Total** **(*n* = 1842)** ***n* (%)**	***x^2^***	***p***
**Willing to get vaccinated (out of unvaccinated)**								
	Highly & somewhat unlikely	50 (10.9)	11 (4.5)	44 (7.2)	48 (9.2)	153 (8.3)	28.02	<0.001
	May be	120 (26.0)	66 (26.8)	104 (17.1)	117 (22.2)	407 (22.1)		
	Highly & somewhat likely	291 (63.1)	169 (68.7)	461 (75.7)	361 (68.6)	1282 (69.6)		

**Table 6:** Positive reasons (factors) for vaccination.

Positive		Thailand (*n* = 461) *n* (%)	Indonesia (*n* = 246) *n* (%)	Philippines (*n* = 609) *n* (%)	Vietnam (*n* = 526) *n* (%)	*x^2^*	*p*
**Enabling environment**							
It’s free							
	Not selected	284 (61.6)	175 (71.1)	437 (71.8)	433 (82.3)	52.75	<0.001
	Selected	177 (38.4)	71 (28.9)	172 (28.2)	93 (17.7)		
Vaccine site is convenient/nearby							
	Not selected	404 (87.6)	208 (84.5)	582 (95.6)	473 (89.9)	32.90	<0.001
	Selected	57 (12.4)	38 (15.5)	27 (4.4)	53 (10.1)		
It is easy to make an appointment							
	Not selected	425 (92.2)	222 (90.2)	599 (98.4)	503 (95.6)	33.94	<0.0001
	Selected	36 (7.8)	24 (9.8)	10 (1.6)	23 (4.4)		
My employer provides paid time off to take it							
	Not selected	444 (96.3)	232 (94.3)	573 (94.1)	503 (95.6)	3.44	0.33
	Selected	17 (3.7)	14 (5.7)	36 (5.9)	23 (4.4)		
**Vaccine effect and safety**							
Vaccines are safe and effective							
	Not selected	303 (65.7)	170 (69.1)	418 (68.6)	251 (47.7)	64.91	<0.001
	Selected	158 (34.3)	76 (30.9)	191 (31.4)	275 (52.3)		
Enough time has passed since people started taking it							
	Not selected	417 (90.5)	221 (89.8)	576 (94.6)	499 (94.9)	13.51	0.004
	Selected	44 (9.5)	25 (10.2)	33 (5.4)	27 (5.1)		
Experts say it is safe and effective							
	Not selected	354 (76.8)	167 (67.9)	376 (61.7)	382 (72.6)	21.54	<0.001
	Selected	107 (23.2)	79 (32.1)	233 (38.3)	144 (27.4)		
Recommended by a physician/doctor							
	Not selected	378 (82.0)	196 (79.7)	508 (83.4)	464 (88.2)	11.86	<0.00
	Selected	83 (18.0)	50 (30.3)	101 (16.6)	62 (11.8)		
My loved ones recommended that I take it							
	Not selected	433 (93.9)	213 (86.6)	569 (93.4)	483 (91.8)	14.05	0.003
	Selected	28 (6.1)	33 (13.4)	40 (6.6)	43 (8.2)		
People I know took it							
	Not selected	436 (94.6)	218 (88.6)	577 (94.8)	487 (92.6)	12.24	0.01
	Selected	25 (5.4)	28 (11.4)	32 (5.2)	39 (7.4)		
Leaders I trust took it							
	Not selected	432 (93.7)	219 (89.0)	588 (96.5)	476 (90.5)	23.50	<0.001
	Selected	29 (6.3)	27 (11.0)	21 (3.5)	50 (9.5)		
**Risk Aversion**							
I’m afraid of getting COVID 19							
	Not selected	280 (60.7)	178 (72.4)	353 (58.0)	252 (47.9)	44.08	<0.001
	Selected	181 (39.3)	68 (27.6)	256 (42.0)	274 (52.1)		
Vaccines will keep my loved ones safe from COVID 19							
	Not selected	259 (56.2)	146 (59.4)	271 (44.5)	294 (55.9)	25.19	<0.001
	Selected	202 (43.8)	100 (40.6)	338 (55.5)	232 (44.1)		
**Lifestyle**							
To return to my previous lifestyle							
	Not selected	326 (70.7)	212 (86.2)	476 (78.2)	427 (81.2)	27.10	<0.001
	Selected	135 (29.3)	34 (13.8)	133 (21.8)	99 (18.8)		
To return to work/school							
	Not selected	415 (90.0)	207 (84.2)	513 (84.2)	451 (85.7)	8.53	0.04
	Selected	46 (10.0)	39 (15.8)	96 (15.8)	75 (14.3)		
**Social Norm**							
My employer requires me to take it							
	Not selected	428 (92.8)	232 (94.3)	533 (87.5)	488 (92.8)	16.50	0.001
	Selected	33 (7.2)	14 (5.7)	76 (12.5)	38 (7.2)		
Others							
	Not selected	457 (99.1)	245 (99.6)	605 (99.3)	536 (100.0)	4.33	0.23
	Selected	4 (0.9)	1 (0.4)	4 (0.7)	0 (0.0)		

**Table 7:** Negative reasons (factors) for vaccination.

Negative		Thailand (*n* = 461) *n* (%)	Indonesia (*n* = 246) *n* (%)	Philippines (*n* = 609) *n* (%)	Vietnam (*n* = 526) *n* (%)	*x^2^*	*p*
**Enabling environment**							
I do not know when and where to get it							
	Not selected	409 (88.7)	179 (72.8)	523 (85.9)	372 (70.7)	72.51	<0.001
	Selected	52 (11.3)	67 (27.2)	86 (14.1)	154 (29.3)		
I do not have the ability to get an appointment							
	Not selected	417 (90.5)	201 (81.7)	541 (88.8)	442 (84.0)	16.77	0.001
	Selected	44 (9.5)	45 (18.3)	68 (11.2)	84 (16.0)		
I cannot access an appointment time outside of my working hours							
	Not selected	431 (93.5)	208 (84.5)	561 (92.1)	477 (90.7)	17.04	0.001
	Selected	30 (6.5)	38 (15.5)	48 (7.9)	49 (9.3)		
I do not trust the sites where vaccines are being administered							
	Not selected	418 (90.7)	217 (88.2)	502 (82.4)	460 (87.5)	16.68	0.001
	Selected	43 (9.3)	29 (11.8)	107 (17.6)	66 (12.5)		
**Vaccine effect and safety**							
Possible side-effects							
	Not selected	111 (24.1)	115 (46.7)	164 (26.9)	234 (44.5)	77.51	<0.001
	Selected	350 (75.9)	131 (53.3)	445 (73.1)	292 (55.5)		
Not enough people have taken the vaccine							
	Not selected	424 (92.0)	206 (83.7)	494 (81.1)	449 (85.4)	25.48	<0.001
	Selected	37 (8.0)	40 (16.3)	115 (18.9)	77 (14.6)		
Approvals and clinical trials were too fast							
	Not selected	398 (86.3)	206 (83.7)	439 (72.1)	465 (88.4)	61.17	<0.001
	Selected	63 (13.7)	40 (16.3)	170 (27.9)	61 (11.6)		
Not enough evidence that it prevents COVID 19							
	Not selected	300 (65.1)	187 (76.0)	384 (63.1)	398 (75.7)	29.90	<0.001
	Selected	161 (34.9)	59 (24.0)	225 (36.9)	128 (24.3)		
Not enough time since people started taking it							
	Not selected	425 (92.2)	214 (87.0)	539 (88.5)	432 (82.1)	23.60	<0.001
	Selected	36 (7.8)	32 (13.0)	70 (11.5)	94 (17.9)		
Vaccines are not safe or effective in general							
	Not selected	174 (37.7)	214 (87.0)	499 (81.9)	460 (87.5)	396.75	<0.001
	Selected	287 (62.3)	32 (13.0)	110 (18.1)	66 (12.5)		
Storage of the vaccine is concerning							
	Not selected	416 (90.2)	210 (85.4)	543 (89.2)	414 (78.7)	35.12	<0.001
	Selected	45 (9.8)	36 (14.6)	66 (10.8)	112 (21.3)		
It contained human stem cells in its development							
	Not selected	429 (93.1)	224 (91.1)	584 (95.9)	475 (90.3)	15.02	0.002
	Selected	32 (6.9)	22 (8.9)	25 (4.1)	51 (9.7)		
Clinical trials did not include adequate diversity or enough people of color							
	Not selected	400 (86.8)	208 (84.5)	491 (80.6)	445 (84.6)	7.88	0.05
	Selected	61 (13.2)	38 (15.5)	118 (19.4)	81 (15.4)		
I am concerned it will impact my fertility or pregnancy							
	Not selected	418 (90.7)	212 (86.2)	565 (92.8)	423 (80.4)	45.11	<0.001
	Selected	43 (9.3)	34 (13.8)	44 (7.2)	103 (19.6)		
Others							
	Not selected	448 (97.2)	242 (98.4)	594 (97.5)	516 (98.1)	1.51	0.68
	Selected	13 (2.8)	4 (1.6)	15 (2.5)	10 (1.9)		
**Risk Aversion**							
I’m not afraid of getting COVID 19							
	Not selected	440 (95.4)	225 (91.1)	560 (91.9)	498 (94.7)	8.86	0.03
	Selected	21 (4.6)	22 (8.9)	49 (8.1)	28 (5.3)		
I already had COVID 19							
	Not selected	452 (98.1)	229 (93.1)	603 (99.0)	512 (97.3)	26.02	<0.001
	Selected	9 (1.9)	17 (6.9)	6 (1.0)	14 (2.7)		
Religious concerns							
	Not selected	455 (98.7)	229 (93.1)	588 (96.5)	501 (92.3)	16.10	0.001
	Selected	6 (1.3)	17 (6.9)	21 (3.5)	25 (4.7)		

**Table 8:** Binomial logistic regression of willingness for vaccine with sociodemographic factors.

		Thailand		Indonesia		Philippines		Vietnam	
		AOR (95% CI)	*p*	AOR (95% CI)	*p*	AOR (95% CI)	*p*	AOR (95% CI)	*p*
**Age**									
	≤34	1		1		1		1	
	>34	1.20 (0.79–1.83)	0.38	2.35 (1.20–4.59)	<0.05	1.27 (0.82–1.98)	0.28	0.50 (0.31–0.78)	<0.05
**Sex**									
	Male	1		1		1		1	
	Female	0.70 (0.47–1.05)	0.09	0.87 (0.48–1.60)	0.66	0.96 (0.65–1.42)	0.86	1.34 (0.90–2.02)	0.15
**Monthly household income**									
	Below average	1		1		1		1	
	Average	1.43 (0.85–2.39)	0.18	1.73 (0.74–4.07)	0.21	0.96 (0.54–1.70)	0.89	0.81 (0.42–1.54)	0.52
	Above average	0.89 (0.52–1.52)	0.67	1.05 (0.5–2.14)	0.90	1.20 (0.67–2.13)	0.55	1.87 (1.10–3.20)	<0.05
**Income decreased due to COVID-19**									
	No	1		1		1		1	
	Yes	0.90 (0.59–1.38)	0.63	1.97 (1.08–3.60)	<0.05	0.88 (0.59–1.31)	0.52	1.47 (1.00–2.25)	0.07
**Education**									
	Up to secondary	1		1		1		1	
	Tertiary and above	2.25 (1.31–3.87)	<0.05	2.46 (1.3–4.67)	<0.05	1.60 (0.84–3.05)	0.15	1.63 (0.81–3.27)	0.17
**Employment**									
	Un-employed	1		1		1		1	
	Retired or homemaker	5.19 (1.30–0.65)	<0.05	1.47 (0.18–11.84)	0.72	0.60 (0.21–1.73)	0.35	2.16 (0.24–19.50)	0.49
	Part-time	0.89 (0.35–2.26)	0.79	0.80 (0.22–2.97)	0.74	1.20 (0.57–2.50)	0.64	2.01 (0.58–7.02)	0.27
	Full-time or business	1.68 (0.71–4.01)	0.24	0.99 (0.27–3.63)	0.99	1.25 (0.64–2.44)	0.51	4.43 (1.30–15.15)	<0.05
**Family type**									
	Living alone	1							
	Living with friends/partner/spouse with or without children	1.19 (0.66–2.18)	0.56	0.80 (0.28–2.32)	0.68	1.08 (0.54–2.19)	0.82	0.86 (0.43–1.71)	0.67
	Multi-generation	1.10 (0.60–2.03)	0.76	1.03 (0.33–3.18)	0.96	1.10 (0.54–2.23)	0.80	0.39 (0.20–0.78)	<0.05

**Table 9:** Binomial logistic regression of willingness for vaccine with virus perception.

		Thailand		Indonesia		Philippines		Vietnam	
		AOR (95% CI)	*p*	AOR (95% CI)	*p*	AOR (95% CI)	*p*	AOR (95% CI)	*p*
**Confidence in Knowledge**									
**I am well read and know a lot about the coronavirus**									
	Strongly & somewhat disagree	1		1		1		1	
	Neither agree nor disagree	1.41 (0.44–4.43)	0.56	0.85 (0.21–3.48)	0.82	0.47 (0.20–1.12)	0.09	0.30 (0.09–0.99)	0.05
	Strongly & somewhat agree	2.07 (0.92–4.65)	0.08	1.04 (0.28–3.87)	0.96	0.73 (0.39–1.37)	0.33	0.82 (0.31–2.19)	0.69
**Concerns about CVID-19 health risk**									
**The coronavirus is similar to the regular flu and has similar death rates**									
	Strongly & somewhat agree	1		1		1		1	
	Neither agree nor disagree	1.07 (0.52–2.19)	0.86	0.74 (0.28–1.98)	0.55	0.45 (0.24–0.82)	<0.05	0.72 (0.34–1.51)	0.39
	Strongly & somewhat disagree	1.41 (0.86–2.28)	0.17	0.79 (0.38–1.65)	0.53	0.96 (0.61–1.52)	0.88	1.13 (0.69–1.85)	0.62
**People of all ages are dying from the coronavirus**									
	Strongly & somewhat disagree	1		1		1		1	
	Neither agree nor disagree	1.35 (0.69–2.65)	0.38	1.22 (0.42–3.57)	0.71	2.32 (0.95–5.68)	0.07	0.62 (0.23–1.66)	0.34
	Strongly & somewhat agree	1.16 (0.71–1.90)	0.56	1.51 (0.59–3.89)	0.39	1.77 (1.00–3.13)	<0.05	1.65 (0.77–3.55)	0.20
**The world is in serious danger due to the coronavirus**									
	Strongly & somewhat disagree	1		1		1		1	
	Neither agree nor disagree	0.51 (0.15–1.74)	0.28	3.26 (0.78–13.58)	0.11	1.19 (0.40–3.57)	0.75	0.63 (0.12–3.39)	0.59
	Strongly & somewhat agree	0.66 (0.27–1.64)	0.37	8.28 (2.23–30.74)	<0.05	3.22 (1.38–7.52)	<0.05	0.89 (0.23–3.45)	0.87
**I am safe from the virus where I live, but not the rest of world**									
	Strongly & somewhat agree	1		1		1		1	
	Neither agree nor disagree	0.53 (0.22–1.30)	0.17	0.63 (0.28–1.40)	0.26	0.52 (0.31–0.88)	<0.05	1.61 (0.91–2.87)	0.10
	Strongly & somewhat disagree	0.59 (0.34–1.04)	0.07	1.18 (0.53–2.64)	0.69	0.88 (0.55–1.42)	0.61	1.00 (0.64–1.57)	0.99
**Most concerned about older relatives/parents**									
	Strongly & somewhat disagree	1		1		1		1	
	Neither agree nor disagree	2.12 (0.55–8.23)	0.28	3.98 (0.91–17.28)	0.07	1.49 (0.31–7.08)	0.62	0.73 (0.25–2.16)	0.58
	Strongly & somewhat agree	0.95 (0.37–2.41)	0.91	4.47 (1.28–15.59)	<0.05	0.98 (0.47–2.01)	0.95	0.89 (0.35–2.24)	0.80
**Financial Concern**									
**Current pandemic economic is worse than that of Global Financial Crisis of 2008**									
	Strongly & somewhat disagree	1		1		1		1	
	Neither agree nor disagree	1.12 (0.38–3.30)	0.83	1.05 (0.41–2.70)	0.91	1.35 (0.57–3.19)	0.49	1.11 (0.53–2.29)	0.79
	Strongly & somewhat agree	0.71 (0.28–1.78)	0.46	1.55 (0.67–3.60)	0.31	1.57 (0.73–3.34)	0.24	1.33 (0.68–2.61)	0.40
**I am not financially secure because of the pandemic**									
	Strongly & somewhat disagree	1		1		1		1	
	Neither agree nor disagree	1.08 (0.36–3.20)	0.89	0.24 (0.07–0.78)	<0.05	1.59 (0.77–3.29)	0.21	1.14 (0.63–2.07)	0.66
	Strongly & somewhat agree	0.69 (0.29–1.66)	0.41	0.38 (0.12–1.15)	0.09	1.09 (0.63–1.89)	0.76	1.28 (0.74–2.21)	0.37

**Table 10:** Binomial logistic regression of willingness for vaccine with positive reasons (factor) to vaccination.

Willingness for vaccination (Unlikely = 0, Likely = 1)		Thailand		Indonesia		Philipines		Vietnam	
Positive		AOR (95% CI )	*p*	AOR (95% CI )	*p*	AOR (95% CI )	*p*	AOR (95% CI)	*p*
**Enabling environment**									
	It’sfree	0.57 (0.37–0.86)	<0.01	0.46 (0.24–0.88)	<0.05	0.64 (0.43–0.97)	<0.05	0.98 (0.59–1.65)	0.96
	Vaccinesiteisconvenient/nearby	0.72 (0.39–1.31)	0.28	0.47 (0.21–1.06)	0.07	0.50 (0.22–1.14)	0.10	1.23 (0.62–2.41)	0.55
	Easytomakeanappointment	0.95 (0.45–2.03)	0.90	0.76 (0.29–1.99)	0.58	0.31 (0.08–1.10)	0.07	2.48 (0.76–8.05)	0.13
	Myemployerprovidespaidtimeofftotakeit	1.43 (0.48–4.28)	0.52	0.64 (0.19–2.19)	0.48	0.70 (0.33–1.51)	0.37	1.33 (0.48–3.71)	0.58
**Vaccine effect and safety**									
	Vaccines are safe and effective	0.68 (0.44–1.03)	0.07	0.86 (0.45–1.65)	0.66	1.40 (0.92–2.14)	0.12	1.14 (0.76–1.72)	0.52
	Enough time has passed since people started taking it	0.34 (0.18–0.67)	<0.01	4.27 (1.16–15.67)	<0.05	0.49 (0.23–1.04)	0.06	0.32 (0.14–0.75)	<0.01
	Experts say it is safe and effective	0.61 (0.38–0.98)	<0.05	1.31 (0.69–2.48)	0.41	1.30 (0.88–1.92)	0.19	0.88 (0.56–1.37)	0.58
	Recommended by a physician/doctor	0.98 (0.58–1.64)	0.94	1.19 (0.57–2.46)	0.64	0.84 (0.51–1.38)	0.49	0.71 (0.39–1.27)	0.25
	My loved ones recommended that I take it	1.07 (0.46–2.46)	0.88	0.74 (0.33–1.67)	0.47	1.39 (0.62–3.13)	0.43	1.40 (0.64–3.05)	0.40
	People I know took it	1.83 (0.69–4.85)	0.22	0.79 (0.31–2.05)	0.64	0.51 (0.24–1.09)	0.08	0.88 (0.41–1.88)	0.74
	Leaders I trust took it	0.96 (0.42–2.15)	0.91	0.57 (0.23–1.40)	0.22	0.91 (0.34–2.45)	0.85	1.10 (0.56–2.17)	0.27
**Risk Aversion**									
	I’m afraid of getting COVID 19	1.38 (0.91–2.09)	0.13	1.43 (0.73–2.79)	0.29	1.97 (1.31–2.95)	<0.01	1.03 (0.69–1.54)	0.88
	Vaccines will keep my loved ones safe from COVID 19	2.49 (1.64–3.80)	<0.001	5.09 (2.47–10.49)	<0.001	1.65 (1.12–2.41)	<0.05	1.49 (0.98–2.25)	0.06
**Lifestyle**									
	To return to my previous lifestyle	1.27 (0.81–1.98)	0.29	0.77 (0.34–1.75)	0.54	1.25 (0.78–2.02)	0.35	0.76 (0.46–1.25)	0.28
	To return to work/school	1.64 (0.80–3.34)	0.17	0.77 (0.36–1.67)	0.51	0.85 (0.51–1.40)	0.52	1.09 (0.61–1.94)	0.77
**Social Norm**									
	My employer requires me to take it	1.86 (0.79–4.35)	0.16	0.67 (0.19–2.33)	0.53	0.45 (0.27–0.75)	<0.01	0.83 (0.38–1.82)	0.65

**Table 11:** Binomial logistic regression of willingness for vaccine with negative reasons (factor) to vaccination.

Willingness for vaccination (Unlikely = 0, Likely = 1)		Thailand		Indonesia		Philipines		Vietnam	
Negative		AOR (95% CI)	*p*	AOR (95% CI)	*p*	AOR (95% CI)	*p*	AOR (95% CI)	*p*
**Enabling environment**									
	I do not know when and where to get it	0.84 (0.45–1.57)	0.58	1.35 (0.68–2.65)	0.39	1.42 (0.80–2.52)	0.23	1.05 (0.68–1.63)	0.82
	I do not have the ability to get an appointment	3.85 (1.61–9.20)	<0.01	1.01 (0.48–2.14)	0.97	3.36 (1.49–7.59)	<0.01	1.29 (0.72–2.29)	0.39
	I cannot access an appointment time outside of my working hours	2.36 (0.92–6.04)	0.07	1.25 (0.56–2.83)	0.58	1.60 (0.72–3.55)	0.24	1.38 (0.68–2.83)	0.37
	I do not trust the sites where vaccines are being administered	1.08 (0.55–2.13)	0.82	0.25 (0.10–0.62)	<0.01	1.03 (0.63–1.70)	0.90	1.14 (0.63–2.07)	0.67
**Vaccine effect and safety**									
	Possible side-effects	0.66 (0.40–1.07)	0.09	0.90 (0.49–1.65)	0.73	0.86 (0.56–1.33)	0.50	0.53 (0.34–0.80)	<0.01
	Not enough evidence that it prevents COVID 19	0.54 (0.36–0.83)	<0.01	1.17 (0.58–2.35)	0.67	0.58 (0.39–0.85)	<0.01	0.70 (0.45–1.11)	0.13
	Approvals and clinical trials were too fast	2.11 (1.11–4.03)	<0.05	2.29 (0.95–5.51)	0.07	0.69 (0.46–1.04)	0.08	1.07 (0.57–2.01)	0.83
	Not enough time since people started taking it	0.80 (0.38–1.68)	0.56	1.04 (0.44–2.45)	0.93	0.95 (0.53–1.71)	0.88	0.84 (0.51–1.40)	0.51
	Not enough people have taken the vaccine	1.99 (0.90–4.41)	0.09	0.62 (0.28–1.34)	0.23	0.76 (0.48–1.20)	0.24	0.84 (0.4–1.49)	0.55
	Vaccines are not safe or effective in general	0.43 (0.28–0.66)	<0.001	0.77 (0.33–1.80)	0.54	0.57 (0.36–0.90)	<0.05	0.97 (0.53–1.79)	0.93
	Storage of the vaccine is concerning	0.72 (0.37–1.40)	0.33	1.59 (0.67–3.75)	0.29	1.24 (0.66–2.33)	0.50	1.67 (0.99–2.83)	0.06
	It contained human stem cells in its development	2.14 (0.88–5.21)	0.09	0.96 (0.33–2.79)	0.95	1.30 (0.47–3.59)	0.61	3.92 (1.60–9.60)	<0.01
	Clinical trials did not include adequate diversity or enough	1.18 (0.65–2.12)	0.59	1.12 (0.49–2.54)	0.78	1.39 (0.83–2.32)	0.20	1.22 (0.70–2.13)	0.48
	I am concerned it will impact my fertility or pregnancy	2.22 (1.03–4.76)	<0.05	1.59 (0.65–3.88)	0.30	0.89 (0.44–1.79)	0.74	1.32 (0.77–2.24)	0.31
**Risk Aversion**									
	I’m not afraid of getting COVID 19	0.88 (0.35–2.23)	0.79	0.63 (0.23–1.71)	0.37	2.22 (0.95–3.15)	0.06	1.07 (0.42–2.72)	0.88
	Religious concerns	1.04 (0.18–5.96)	0.96	0.45 (0.15–1.39)	0.17	1.46 (0.48–4.47)	0.51	1.04 (0.40–2.68)	0.94
	I already had COVID-19	4.92 (0.58–41.64)	0.14	1.47 (0.43–5.09)	0.54	1.45 (0.16–13.08)	0.74	0.55 (0.17–1.78)	0.32
